# Allostasis and the origins of adult-onset diabetes

**DOI:** 10.1007/s00125-019-05048-9

**Published:** 2019-12-07

**Authors:** R. David Leslie, Tanwi Vartak

**Affiliations:** Blizard Institute, Barts and the London School of Medicine and Dentistry, Queen Mary University of London, 4 Newark Street, London, E1 2AT UK

**Keywords:** Autoantibodies, Autoimmunity, C-peptide, Diabetes, Diabetes heterogeneity, Diabetes risk, Genetics

## Abstract

Physiological plasticity enables homeostasis to be maintained in biological systems, but when such allostasis fails, then disease can develop. In a new population-based study by Rolandsson et al (10.1007/s00125-019-05016-3), autoimmunity, defined by an immunogenotype, predicted adult-onset non-insulin requiring diabetes. Type 1 diabetes is no longer viewed as a disease confined to children, with a significant proportion, maybe the majority, presenting in adulthood. Such cases masquerade as type 2 diabetes and their identification has clinical utility. Nevertheless, in this study, autoimmunity had a limited effect on the overall risk of adults developing diabetes.

Epidemics in the 19th century were, in the main, due to infectious diseases; diabetes was different. In 1888 Etienne Lancereaux, a French physician, believed that individuals with diabetes were either thin (‘maigre’) or fat (‘gras’) [1]. More precisely, the latter were invariably rich and fat. Adult-onset diabetes was considered a disease of civilisation, secondary to poor digestion and bad nerves, but a homogeneous disease, nonetheless, with a homogeneous outcome. In contrast people with ‘diabete maigre’ were invariably thin, young, desperately ill and, within a year, dead. The introduction of insulin in 1921 commuted the death sentence to a life sentence but emphasised that self-evident distinction between the insulin-dependent form of diabetes and everything else, then called non-insulin-dependent diabetes, now type 2 diabetes. Ascribing the modern global epidemic of adult-onset diabetes to a maladaptation to civilisation did not, until recently, alter the perspective that the disease could be lumped under one umbrella with a common pathogenesis and uniform therapeutic pathway.

## Diabetes as a binary disease

**Fig. 1:**
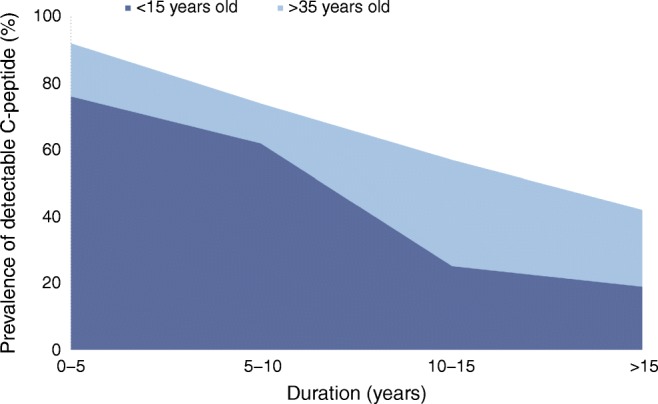
Prevalence of detectable C-peptide by age at diabetes onset and disease duration (in years). The plot shows the prevalence of detectable C-peptide by age at onset and duration (years) of the disease. The prevalence of C-peptide was 19% in those who were <15 years old at disease onset and with a disease duration of ≥15 years vs 72% for those who were >35 years old at disease onset, with a disease duration of <15 yeasrs. The diffrence in detectable C-peptide both at baseline and years after diagnosis between younger patients (<15 years old) and older patients (>35 years old) is clear since differences in persistent C-peptide relate to disease age at onset and duration, especially the former. Data from Mckeigue et al [21]

That binary perspective of diabetes changed as we engaged with maturity onset diabetes of the young (MODY) and adult-onset autoimmune diabetes, also called latent autoimmune diabetes in adults (LADA) [2]. Awareness that the patient in front of you, young or old, lean or fat, could have different forms of diabetes, has pushed physicians away from simple clinical assessment into the realm of precision medicine. For the patient with adult-onset diabetes, three features, aside from demographics, have provided traction in dissecting the nature of this disease process: genetics, including genetic risk scores (GRS); immunology, in the form of diabetes autoantibodies, including autoantibodies to GAD65 (GADA), and serum C-peptide as a proxy for insulin secretion (Fig. 1) [2, 3].

## GADA and GRS predicts adult-onset diabetes

**Fig. 2:**
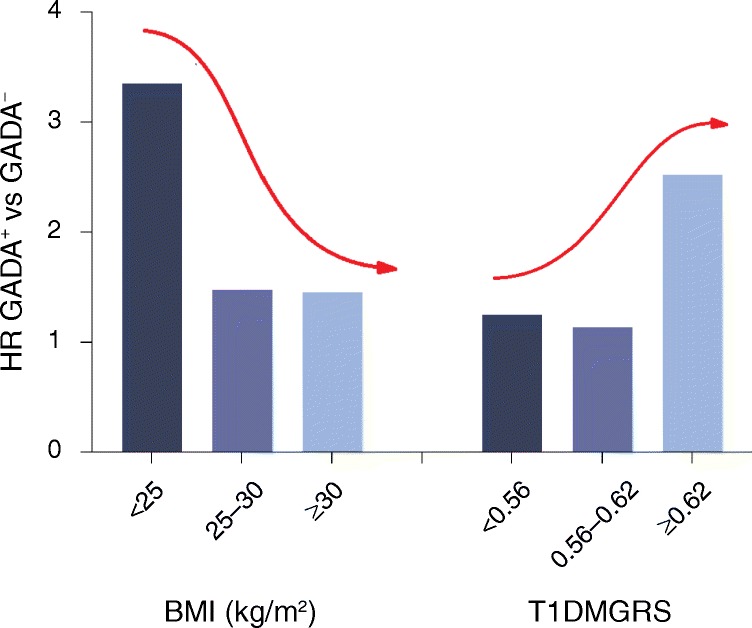
HRs for incident diabetes comparing GADA^+^ with GADA^–^ cases within categories of tertiles of BMI and type 1 diabetes mellitus GRS (T1DMGRS). The plot shows the HR for incident diabetes comparing GADA^+^ with GADA^–^. High HR has been associated with low BMI (<25 kg/m^2^); the opposite trend is seen with a high HR for values shown for T1DMGRS. This shows that GADA^+^ cases tend to be both genetically and phenotypically closer to type 1 diabetes than type 2 diabetes. Data from Rolandsson et al [4]

In this issue of *Diabetologia*, Rolandsson and colleagues have leveraged two of these three markers, i.e. GADA and GRS, in European Prospective Investigation into Cancer and Nutrition (EPIC), a large cohort of individuals (over 340,000 participants over 40 years of age and free of known diabetes at baseline), followed for 10 years to determine the potential for these markers to identify those at risk of diabetes [4]. For those with GADA initially, there was indeed an increased risk that diabetes would develop during follow-up (HR 1.78 for GADA positive vs GADA negative). Moreover, the GRS for type 1 diabetes, but not for type 2 diabetes, was associated with GADA in incident cases (OR 1.97) (Fig. 2). The GRS was calculated by multiplying the number of disease risk-increasing gene alleles by the natural log of OR at each locus, then summing these ORs for each individual. This risk for developing diabetes in the EPIC cohort was further increased to 3.2 by combining GADA positivity with the top tertile of type 1 diabetes risk according to the GRS (Fig. 2). The authors, in concluding that incident adult-onset diabetes has an element of autoimmunity, proposed that the present sub-classification of diabetes in adulthood should be re-evaluated.

In one sense, we could have anticipated these results. GADA are known to appear largely within the first decade of life and predict diabetes in children [5]. However, there have only been a couple of previous similar prospective studies reporting that GADA predicted adult-onset diabetes. In the Botnia Study, GADA (vs no GADA) predicted non-insulin-requiring diabetes (HR 4.9) [6], while in the Nord-Trøndelag Health Study (HUNT), even evanescent GADA predicted diabetes and these diabetes patients tended to be younger, with lower C-peptide and higher HbA_1c_ [7, 8]. As illustrated by this present large study from EPIC, the percentage with GADA was small, and so the percentage with GADA who developed diabetes was small. It seems reasonable to conclude from these studies that autoimmunity does not play a major role in the development of adult-onset diabetes among older adults. Certainly, in population-based studies of adults with diabetes the frequency of GADA is low, as with this present European study, while in clinic-based studies, that GADA frequency tends to be 5–10%, whether in China or Europe [4, 9–11]. Prospective data from HUNT indicated that incident diabetes cases, who were overweight with a family history of diabetes plus GADA positivity, as compared with GADA negative cases, had an OR for diabetes risk of 7.5. By implication, GADA positivity in the general population identifies a small number at risk of late adult-onset autoimmune diseases, that is thyroid autoimmunity and diabetes [12]. For diabetes this risk is higher in overweight individuals with both a family history of diabetes and high GRS for type 1 diabetes.

## Disease identity and uncertainty

At the heart of this discussion is the issue of disease identity. Few common diseases are clinically homogeneous; most show a continuum of clinical changes. Autoimmune diseases are no exception. Type 1 diabetes is also a heterogeneous condition with diverse immunogenetic characteristics and clinical outcomes [2]. This clinical and immunogenetic heterogeneity extends from childhood-onset autoimmune diabetes into adult-onset autoimmune diabetes, including people presenting who do not need insulin therapy [3]. It used to be argued that non-insulin-requiring adult-onset autoimmune diabetes either did not exist or was classic type 1 diabetes caught fleetingly in progression before insulin treatment was required, which would contrast with the clinical spectrum in Hashimoto’s thyroiditis. This argument was based on the specificity of the GADA assay when applied to a general population; in which case a substantial fraction of GADA positive cases would be false positives. But the argument does not extend to positive predictive values, which can be altered by reconfiguring cut-off points in specific cohorts, enriching the cohort for clinical features such as adult-onset diabetes, even altering the assay itself [10, 13]. Nor would it explain the predominance of GADA in all adult-onset diabetes cohorts when other diabetes-associated autoantibodies can be detected, but at far lower frequency, or the marked drop in GADA positivity with increasing age at diagnosis in adults [14]. Nevertheless, there will be a false-positive rate, even with the state-of-the-art GADA assay employed by Rolandsson et al [4], plus an unquantified, likely low, false-negative rate. GADA can be transient and, while invariably predominant, do consistently underestimate the percentage of diabetes cases with diabetes-associated autoantibodies e.g. in a clinic-based adult diabetes cohort, diabetes-associated autoantibodies other than GADA detected an additional 7.3% of autoimmune cases [9, 10]. By implication, there will be a small number of additional individuals with other diabetes-associated autoantibodies that were not assayed.

## Disease risk, scale and certainty

To support the argument that this cohort of adult-onset autoimmune diabetes cases have type 1 diabetes they should have the type 1 diabetes-associated genetic risk including histocompatibility antigen (HLA)-associated risk. Major studies from Europe and China have shown just that [15, 16]. A large European cohort of individuals with LADA who were not treated with insulin at diagnosis showed four genetic signals that achieved genome-wide significance at established type 1 diabetes risk loci (*HLA*, *PTPN22*, *INS* and *SH2B3*) [16]. Indeed, these same four loci were identified in a major Chinese study of individuals with adult-onset type 1 diabetes who were treated with insulin from diagnosis [15]. Despite strong genetic correlations across the whole age range, childhood-onset type 1 diabetes shows a greater genetic load than adult-onset type 1 diabetes, consistent with higher GRS and higher disease concordance rates in twins with childhood-onset diabetes [17]. By implication, a GRS derived from childhood-onset type 1 diabetes will underestimate the numbers of cases with type 1 diabetes genetic risk if applied to an adult cohort [18]. This underestimation, together with the likely reduced sensitivity for GADA using this highly specific GADA assay and the likely failure to identify all autoimmune cases as only GADA were tested means that the actual numbers of autoimmune cases will probably be higher than estimated. That said, attributable risk in this population of adult-onset diabetes with GADA is very low at 1.8%, so even a substantial error would have little impact on the overall risk of autoimmune diabetes in adults aged over 40 years [4]. However, the risk of GADA-positive cases developing autoimmune diabetes would likely be much higher in younger adults, as autoimmune diabetes is more prevalent in this age group than in older adults.

Rolandsson et al emphasise the importance of scale in their data and the notion that autoimmune damage could contribute to the development of adult-onset diabetes, whether defined clinically as insulin dependent or non-insulin dependent [4]. Certainly, individuals with adult-onset diabetes who have a high type 1 diabetes GRS and high-titre GADA are at risk of progressing to insulin dependence [19]. This risk falls as the GRS falls [20]. C-peptide levels vary across the age range and with disease duration, even for individuals diagnosed clinically and immunologically with type 1 diabetes (Fig. 1) [21]. Older GADA-positive patients, as studied here, with moderate GRS for type 1 diabetes may clinically resemble patients with type 2 diabetes in that they could have substantial C-peptide. A prospective study of C-peptide in older low-titre GADA-positive Chinese patients (vs high titre) revealed a natural history indistinguishable from type 2 diabetes over a number of years [22]. In a large Chinese population-based adult cohort, ascertained from a notably younger age than in the present study (i.e. over 20 years), the standardised prevalence rate of autoimmune diabetes was 6.0% in adults with diabetes who did not initially require insulin, which corresponds to six million adults in China with a form of autoimmune diabetes that is initially non-insulin requiring [9].

## Allostasis and tipping points

As insulin secretory capacity is compromised, perhaps by an autoimmune process, so pathways that maintain glucose homeostasis and glucose disposition should adjust the levels of insulin secretion to insulin sensitivity. Allostasis, the ability to adapt to maintain glucose homeostasis, must be compromised for this homeostasis to be lost with ensuing dysglycaemia. Taken together, the present data can be viewed from a dual perspective. On one hand, elements of type 2 diabetes might have an autoimmune basis and from that perspective the authors call for a re-evaluation of the present sub-classification of diabetes in adulthood. The problem with type 2 diabetes is that it lacks a biomarker and is effectively a diagnosis of exclusion [2, 3]. Furthermore, the GRS for type 2 diabetes is in general very low and many thousands of patients need to be studied to define differences from a ‘normal’ population. Quite what constitutes a ‘normal’ population is another matter, given the widespread predisposition to dysglycaemia with age. If these GADA-positive autoimmune cases have a form of type 2 diabetes, then they would be expected to have an increased BMI and waist hip ratio, whereas the converse is true (Fig. 2) [4]. From another perspective, the data could be seen to validate the hypothesis that autoimmune diabetes is a spectrum ranging from childhood-onset type 1 diabetes to adult-onset non-insulin-dependent diabetes masquerading as type 2 diabetes [2]. Certainly, the higher GRS for type 1 diabetes and leaner habitus of the GADA-positive individuals is consistent with this proposal (Fig. 2) [4]. Either way, the argument is not futile as there is clinical utility in the identification of autoimmune diabetes. Such patients tend to have a more dynamic clinical history requiring more frequent clinical review. In addition, they should probably not be treated with sulfonylureas, they should be investigated for other forms of autoimmunity and they are at particular risk of hyperglycaemia and progression to insulin therapy [2, 23–25].

In short, the autoimmune process is associated with diabetes, but that risk is a continuum, just as glucose and glucose disposition, insulin secretion and insulin sensitivity represent continua. For the physician, biomarkers are merely guides to management but will surely help a more nuanced approach to the patient sitting in front of you.

## Abbreviations

EPIC: European Prospective Investigation into Cancer and Nutrition
GADA: GAD65 autoantibodies
GRS: Genetic risk score
HUNT: Nord-Trøndelag Health Study
LADA: Latent autoimmune diabetes in adults
